# The level of ROCK1 and ROCK2 in patients with pulmonary hypertension in plateau area

**DOI:** 10.1038/s41598-018-27321-4

**Published:** 2018-06-19

**Authors:** Bing Liu, Rong Chang, Zhili Duan, Xiaofei Zhang, Yusong Shen, Xiangbo Liu, Jinchun Wu, Yajun Tuo, Junming Luo

**Affiliations:** 1 Department of Cardiology, Qinghai Provincial People’s Hospital, Xining, 810007 China; 2 Department of Pathology, Qinghai Provincial People’s Hospital, Xining, 810007 China; 3 Department of Pneumology, Qinghai Provincial People’s Hospital, Xining, 810007 China

## Abstract

Pulmonary hypertension (PH) is defined as the mean pulmonary artery pressure (mPAP) ≥25 mmHg under the sea level in resting state. ROCK1 and ROCK2 can be combined to cause the damage of vascular endothelial function. To explore the differences of ROCK1 and ROCK2 in subjects with pulmonary hypertension or normal pulmonary artery pressure in plateau area, and to further understand the mechanism of Rho/rho-kinase pathway activation for promoting pulmonary hypertension, we collected 64 patients with pulmonary hypertension and 87 normal pulmonary artery healthy controls. All subjects were hospitalized in Cardiology or Respiration Department of Qinghai Provincial Peoples’ Hospital from December 2016 to June 2017. The pulmonary artery systolic pressure was measured by Doppler ultrasound, and serum ROCK1 and ROCK2 levels were tested by enzyme linked immunosorbent assay (ELISA). We found that the serum ROCK2 concentration in the pulmonary hypertension group was significantly higher than that in the control group, but serum ROCK1 level had no significant difference. ROCK2 plays a leading role in pulmonary hypertension in the plateau region, so selective ROCK2 inhibitors will be more effective in improving pulmonary hypertension.

## Introduction

Pulmonary hypertension (PH) is defined as the mean pulmonary artery pressure (mPAP) ≥25 mmHg under the sea level in resting state. The classic measurement of the mPAP as a gold standard is by cardiac catheter of right heart. PH can be diagnosed by pulmonary artery systolic pressure (PASP). The PASP should be calculated with the three tips regurgitation velocity and the Bernoulli equation. In the absence of obstruction of the right ventricular outflow tract, the PASP is equal to the right ventricular systolic pressure (RVSP); Armstrong DWJ claimed that RVSP = 4V^2^ + RAP by the Bernoullic equation, with V and RAP representing the peak of tricuspid reflux velocity and mean right atrial pressure respectively, and RAP is 10 mmHg in general^1^. Taylor J raised that V > 3.4 m/s (namely PASP > 50 mmHg) was diagnosed as pulmonary hypertension^2^. According to the different causes, pulmonary hypertension can be classified into five categories: arterial pulmonary hypertension, left heart disease related to pulmonary hypertension, lung disease and (or) of hypoxic pulmonary hypertension, chronic thromboembolic pulmonary hypertension, idiopathic pulmonary arterial hypertension; The second and third pulmonary arterial pressures presented different dynamic characteristics^3^.

The Qinghai Province located in Qinghai-Tibet plateau, with attitude of all cities and countryside affiliated ranging from 1500 m to 5000 m, owns unique climate characterized by hypoxia, low pressure and severe coldness, and which can cause pulmonary hypertension and other diseases.

ROCKs (Human Rho Associated Coiled Coil Containing Protein Kinase) are the downstream signal of RhoGTP, which is a kind of serine/threonine kinase, there are two homologous isomers: ROCK1 and ROCK2. The ROCK1 gene is located on chromosome 18 and consists of 1354 amino acids, mainly expressed in blood cells and thymus tissue, while the ROCK2 gene is located on chromosome 2, a polypeptide composed of 1388 amino acids, which is highly expressed in the cerebrovascular tissue cells, ROCK1 and ROCK2 share 65% of amino acid sequence as well as 92% of kinase domains. Both DNA dissolve temperature have no obvious difference, 78.5 °C and 78 °C respectively^4,5^, but they play different roles in our bodies. ROCK1 and ROCK2 all contain three domains: N-terminal kinase binding domain, curly helical region (including Rho binding domain), C-terminal domain (rich in cysteine).

In normal circumstances, the C-terminal domain inhibits the activity of the N-terminal domain, causing ROCK without activation. In the pathological state such as hypoxia, ROCKs can be activated by RhoGTP and Rho combining domain or caspase-3 resection C-terminal and so on to remove the inhibition of C-terminal to N-terminal. Previous studies have shown that ROCK1 activated can participate in myocardial cell apoptosis, cardiac fibrosis lead to symptoms and signs of cardiac dysfunction, as well as which also participate in vascular smooth muscle cell proliferation, inflammatory adhesion factor expression, leukocyte infiltration and so on^4,6^, and ROCK2 can promote the migration of vascular smooth muscle cells and mitosis, cell adhesion, inflammatory cell recruitment, extracellular matrix abnormal deposition, myogenic reaction, etc^7,8^, while both of them induce pulmonary vasoconstriction and remodeling, ventricular hypertrophy and right ventricular dysfunction, resulting in pulmonary hypertension. In addition, in course of diabetes, ROCK1 and ROCK2 can be combined to cause the damage of vascular endothelial function^9^. In previous articles, we described the pathophysiological changes of pulmonary hypertension, which not only manifested pulmonary vasoconstriction, pulmonary vascular remodeling, but also included the right cardiac structure and functional changes. A large number of animal experiments demonstrated that the Rho/rho-kinase pathway was involved in the progression of this disease. Currently, studies on the relationship between pulmonary hypertension and ROCK1 and ROCK2 were limited to lung tissue level. However, the study of pulmonary hypertension in the plateau region is limited, and the detection of serum ROCK1 and ROCK2 in pulmonary hypertension patients is much more convenient. To compare the levels of serum ROCK1, ROCK2 between the pulmonary hypertension group and control group is still necessary to clear their role in pulmonary hypertension in plateau area, as well as understand the pathogenesis of pulmonary hypertension in plateau area.

## Results

In the Table 1, the composition of hypertension, the body mass index and systolic blood pressure as well as diastolic blood pressure in the control group were significantly higher than those in the pulmonary hypertension group (*P* < 0.05). At the same time, the oxygen satiation in the pulmonary hypertension group was significantly lower (*P* < 0.05), which indicated chronic hypoxia.

**Table 1:** comparison of basic information of pulmonary hypertension and control group.

	Control (n = 87)	Pulmonary Hypertension (n = 64)	*SV*	*P*
Age (y)	67 (53, 74)	66.0 (53.0, 76.8)	−0.290	0.772
Altitude (m)	2261 (2261, 2850)	2261 (2205, 2666)	−1.620	0.105
Residence time(y)	65 (52, 73)	65 (50, 72)	−0.200	0.842
Height (m)	1.65 (1.60, 1.74)	1.65 (1.58, 1.72)	−0.957	0.339
Weight (kg)	66.6 ± 14.0	62.3 ± 13.5	1.903	0.059
BMI (kg/m^2^)	24.63 ± 4.22	22.88 ± 4.17	2.537	0.012
SpO2(%)	91 (88, 94)	86 (80, 92)	−3.698	0.000
SBP (mmHg)	132 (120, 141)	120 (110, 140)	−2.975	0.003
DBP (mmHg)	80 (73, 87)	75 (70, 81)	−2.869	0.004

SBP: systolic blood pressure, DBP: diastolic blood pressure, SpO2: oxygen saturation of fingertip, SV: statistical values.

In Table 2, Statistical difference of significance in disease composition existed in between Pulmonary Hypertension Group and the Control Group (*P* < 0.05). It was obvious that cases of chronic corpulmonale with an approximate ratio of 50% were the most in Pulmonary Hypertension Group,with pulmonary hypertension being the critical link of lung diseases developing into chronic corpulmonale.

**Table 2:** Comparison of disease composition in pulmonary hypertension and control group [n(%)].

Disease Category	Control	Pulmonary Hypertension	*X* ^2^	*P*
Chronic corpulmonale	10(11.49)	32(50.00)		
Coronary heart disease	15(17.24)	9(14.06)		
Dilated cardiomyopathy	5(5.75)	2(3.13)		
Valvular heart disease	2(2.30)	5(7.81)		
Congential cardiovascular disease	5(5.75)	5(7.81)	49.217	0.000
Hypertension	38(43.68)	4(6.25)		
Atrial fibrillation	5(5.75)	3(4.69)		
Pulmonary embolism	0(0)	2(3.13)		
Others	9(10.34)	2(3.13)		

In Table 3, the anterior and posterior diameter of the left atrium of the control group was significantly greater than that in the pulmonary hypertension (*P* > 0.05). While the indexes of the right heart structure and function in the pulmonary hypertension group were significantly higher than that in the control (*P* < 0.05). The increase of the anterior wall thickness of the right ventricle is a characteristic of pulmonary hypertension. As the pressure of pulmonary artery increases, the load on the right heart increases. To ensure normal pulmonary circulation, myocardial cell proliferation, hypertrophy, right atrium anterior and posterior diameter as well as right ventricular outflow tract increased. So, the right heart change is consistent with pulmonary hypertension.

**Table 3:** Comparison of heart color doppler in pulmonary hypertension and control group.

	Control (n = 87)	Pulmonary Hypertension (n = 64)	SV	*P*
LAD (mm)	38 (35, 43)	41 (37, 49)	−2.959	0.003
IVST (mm)	10 (9,11)	10 (9, 11)	−0.451	0.652
LVPWT (mm)	10 (9,11)	10 (9, 11)	−0.518	0.604
LVEDD (mm)	48 (44, 50)	46 (41, 56)	−0.662	0.508
LVESD (mm)	30 (27, 32)	28.5 (25, 37)	−0.570	0.569
LVFS (%)	36 (32, 39)	34 (30, 38)	−1.558	0.119
EF (%)	65 (60, 68)	63 (57, 68)	−1.163	0.245
MPAD (mm)	23 (21, 26)	30 (26, 32)	−6.799	0.000
RAD (mm)	36 (33, 42)	46 (41, 54)	−7.408	0.000
RVD (mm)	21 (20, 23)	27 (21, 29)	−5.337	0.000
RVAW (mm)	5 (5, 5)	5 (5, 6)	−3.098	0.002
RVOT (mm)	31 (30, 34)	36 (32, 40)	−4.992	0.000

LAD: left atrium diameter, IVST: interventricular septum, LVPWT: left ventricular posteriorwall thickness, LVEDD: left ventricular diastolic diameter, LVESD: left ventricular systolic pressure, LVFS: fraction shortening of left ventricular, EF: ejection fraction, MPAD: main pulmonary artery diameter, RAD: right atrium diameter, RVD: right ventricle diameter, RVAW: anterior wall thickness of the right ventricle, RVOT: the diameter of right ventricular outflow tract.

In Table 4, although there was no significant difference in white blood cell count between the two groups (*P* > 0.05), the ratio of neutrophils and white blood cells in the pulmonary hypertension group was significantly higher than control group (*P* < 0.05). Red blood cells and hemoglobin levels in the pulmonary hypertension group slightly decreased, while there was no significant difference (*P* > 0.05). However, the standard deviation and variation of erythrocyte distribution were significantly higher in the pulmonary hypertension group (*P* < 0.05), indicating anemic state, which could further lead to hypoxia. In addition, the ratio of lymphocytes and white blood cells in pulmonary hypertension group was significantly lower (*P* < 0.05).

**Table 4:** Comparison of blood routine results between pulmonary hypertension group and control group.

	Control (n = 87)	Pulmonary Hypertension (n = 64)	SV	*P*
WBC (×10^9^/L)	5.85 (4.62, 6.89)	6.21 (4.55, 7.70)	−0.477	0.633
NEU (%)	60.8 (52.7, 70.0)	69.4 (61.4, 77.9)	−4.164	0.000
LYM (%)	28.2 (19.7, 34.0)	19.1 (12.5, 26.4)	−4.591	0.000
EOS (%)	1.6 (0.8, 2.5)	1.2 (0.5, 2.5)	−0.174	0.861
BASO (%)	0.6 (0.4, 0.8)	0.5 (0.4, 0.8)	−0.206	0.836
MONO (%)	8.0 ± 2.4	8.5 ± 2.0	−1.269	0.207
RBC (×10^12^/L)	4.92 (4.43, 5.45)	4.80 (4.08, 5.77)	−0.460	0.646
HGB (g/L)	152 ± 29	145 ± 32	−0.520	0.646
HCT(%)	45.5 (42.2, 51.3)	45.2 (39.3, 52.1)	−0.828	0.407
MCV (fl)	93.3 (90.5, 95.8)	93.9 (89.6, 98.7)	−1.062	0.288
MCHC (pg/L)	327 (317, 336)	315 (304, 325)	−0.343	0.001
RDWSD (fl)	47.0 (44.4, 51.2)	54.6 (48.3, 62.9)	−4.949	0.000
RDWCV (%)	13.6 (13.0, 15.1)	15.7 (14.0, 19.6)	−4.636	0.000
PLT (×10^9^/L)	153 (104, 215)	153 (115, 187)	−1.332	0.183

WBC: white blood cell, NEU: neutrophilic granulocyte, LYM: lymphocyte, EOS: eosinocyte, BASO: basicyte, MONO: monocyte, RBC: red blood cell, HGB: hemoglobin, HCT: hematocrit, MCV: mean corpuscular volume, MCH: mean corpuscular hemoglobin, MCHC: mean corpuscular hemoglobin concentration, RDWSD: the standard deviation of the distribution of red blood cell, RDWCV: the variation coefficient of the distribution of red blood cells, PLT: platelet, SV: statistical values.

In Table 5, there was no significant difference between glutamate transaminase and glutamate transaminase in the two groups (*P* > 0.05). However, the level of total bilirubin and direct bilirubin in the pulmonary arterial hypertension group was significantly higher than that in the control group (*P* < 0.05), It is suggested that with the occurrence of pulmonary hypertension, liver function injury caused by the right heart failure.

**Table 5:** Comparison of hepatic function between pulmonary hypertension group and control group.

	Control (n = 87)	Pulmonary Hypertension (n = 64)	SV	*P*
ALT (U/L)	22.00 (14.00, 35.00)	18.50 (11.00, 28.75)	−1.682	0.090
AST (U/L)	26.00 (19.00, 33.00)	24.50 (20.00, 37.75)	0.311	0.756
TBil (umol/L)	16.96 (13.48, 23.96)	21.89 (15.14, 34.08)	−2.700	0.007
DBil (umol/L)	3.90 (2.80, 5.80)	7.25 (3.75, 12.08)	−3.998	0.000
IBil (umol/L)	12.78 (9.88, 18.11)	14.35 (9.68, 23.13)	−1.320	0.187
TP (g/L)	66.5 ± 6.7	60.9 ± 7.7	4.785	0.000
ALB (g/L)	37.2 ± 5.0	34.3 ± 5.7	3.258	0.001

ALT: alanine aminotransferase, AST: aspartate aminotransferase, TBil: total bilirubin; DBil: direct bilirubin, IBil: indirect bilirubin, TP: total protein, ALB: albumin, SV: statistical values.

In Table 6, blood urea nitrogen, creatinine, uric acid and cystatin C in the pulmonary hypertension group were significantly higher than those in the control group (*P* < 0.05). The reason may be the right heart failure in patients with pulmonary hypertension. The decrease of the left cardiac blood circulation led to insufficient renal perfusion. Kidney function deteriorates and metabolism decreases, so urea nitrogen, creatinine and uric acid levels were increased. And cystatin C also increases, which reflected the deterioration of renal function.

**Table 6:** Comparison of renal function between pulmonary hypertension and control group.

	Control (n = 87)	Pulmonary Hypertension (n = 64)	SV	*P*
BUN (mmol/L)	5.59 (4.56, 6.84)	7.13 (5.51, 9.59)	−3.502	0.000
Cr (umol/L)	67 (56, 79)	82 (59, 110)	−3.318	0.001
UA (umol/L)	350 (289, 420)	472 (355, 573)	−4.528	0.000
Glu (mmol/L)	5.19 (4.60, 6.30)	5.09 (4.06, 6.50)	−0.766	0.444
CysC (mg/L)	0.83 (0.69, 0.99)	0.99 (0.77, 1.43)	−3.747	0.000
CRP (mg/dL)	0.307 (0.081, 1.139)	1.054 (0.441, 2.343)	−4.012	0.000

BUN: blood urea nitrogen, Cr: creatinine, UA: uric acid, Glu: glucose, CysC: cystain C, CRP: C reactive protein.

In Table 7, the serum ROCK2 level in the pulmonary hypertension group was significantly higher than control (*P* < 0.05). Although the median of serum ROCK1 in the pulmonary hypertension group was higher than control, there was no significant difference (*P* > 0.05).

**Table 7:** Comparison of serum ROCK1 and ROCK2 levels between the pulmonary hypertension group and the control group.

	Control (n = 87)	Pulmonary Hypertension (n = 64)	SV	*P*
ROCK1 (ng/mL)	20.9 (7.7, 36.5)	25.9 (8.9, 35.5)	−0.439	0.661
ROCK2 (pg/mL)	1131.9 (808.7, 1762.6)	1570.5 (974.0, 2244.5)	−2.694	0.007

## Discussion

### ROCK2 plays a leading role in the formation of pulmonary hypertension

Pulmonary hypertension is mainly characterized as pulmonary vasoconstriction, pulmonary vascular remodeling, right ventricular hypertrophy, and right heart failure and so on, its pathogenesis is complicated, especially the ROCK plays a key role in its development. The serum ROCK2 level in the pulmonary hypertension group was significantly higher than that in the control group (*P* < 0.05), while there was no significant difference in serum ROCK1 (*P* > 0.05). Many studies have shown that the Rho/Rho-kinase pathway involved in pulmonary hypertension. Combined with the experimental results in Table 6, we speculate that ROCK2 plays a major role in pulmonary hypertension while ROCK1.

#### ROCK2 promotes pulmonary vasoconstriction

Existing research shows that ROCK2 rather than ROCK1 directly combined with myosin phosphatase binding subunit, which resulted in the increasing of phosphorylation myosin and leading to the pulmonary vasoconstriction. So, it is suggested that ROCK2 plays an important role in pulmonary artery contraction^10,11^.

#### ROCK2 promotes pulmonary vascular remodeling

Vascular smooth muscle cells and vascular endothelial cells are involved in vascular remodeling. Xu *et al*.^12^ used the small interfering RNA of ROCK2 to treat pulmonary smooth muscle cells, and found that the cell survival cycle was shortened, and the cell apoptosis increased. Shimizu T found that the proliferation and migration of PASMCs in ROCK2 overexpression group were significantly higher than that in the lower expression group of ROCK2, while the role of ROCK1 was relatively small^13^. The above studies show that ROCK2 plays an important role in the proliferation of smooth muscle cells. FENG QIAO *et al*.^14^ showed that the activity of endothelial cells decreased after using siRNA to silence the ROCK2 gene. At the same time, they found that under low oxygen, Rho/Rho-kianse pathway was activated, ROCK2 promoted cell division phase G0/G1 phase to S phase by Up-regulated A/D1 cell cycle protein expression to shorten cell cycle and promote endothelial cell proliferation. While excessive proliferation can result in tumor and pleomorphic lesions, which can damage the medial vascular and elevate pulmonary arterial pressure. According to the previous analysis, it is certain that the main function of pulmonary vascular remodeling is ROCK2 rather than ROCK1.

#### ROCK2 promotes right ventricular hypertrophy and right heart failure

Another characteristic of pulmonary arterial hypertension is a decrease in the right ventricular hypertrophy and systolic function, and right heart failure is the leading cause of most of the patients with pulmonary hypertension. In Table 2, we found that the right cardiac structure of patients with pulmonary hypertension was significantly altered, which was in line with pathophysiological changes. Toru Shimizu^15^, treated cardiac fibroblasts with siRNA and found that The expression of tissue growth factor (CTGF) and fibroblast growth factor (FGF2) was decreased after the ROCK2 gene silencing, while myocardial cell proliferation is not obvious. The research confirmed that under the condition of cardiac remodeling or low oxygen, AngII express in the body was increased to mediate the activation of cardiac fibroblasts and increase the expression of FGF2 and CTGF in fibroblasts by TGF-1. The results are consistent with the experimental results in Table 6. With the activation of fibroblasts, type I collagen increased, but matrix proteinase-1 activity decreased, and extracellular collagen deposition caused myocardial cell contraction^15^. Michelle Surma use ROCK1 or ROCK2 loss functions with antioxidant treatment against myocardial toxicity induced by doxorbicin chemotherapy, found that ROCK1 loss function can enhance the effect and ROCK1 and ROCK2 play a role in instability of actin respectively^6^. Again, it strongly proves that the right cardiac dysfunction is related to ROCK2 rather than ROCK1. The serum ROCK2 level in the pulmonary hypertension group was significantly higher than that in the control group (*P* < 0.05). In accordance with the above experiments, we conclude that ROCK2 rather than ROCK1 plays a leading role in the occurrence of pulmonary hypertension by promoting pulmonary vasoconstriction, pulmonary vascular remodeling, right ventricular hypertrophy, right heart failure occurs.

### Chronic inflammatory response can induce pulmonary hypertension

Neutrophils and C-reactive protein were elevated in the pulmonary hypertension group (*P* < 0.05), consistent with the results of Voelkel *et al*. The increase of neutrophils can enhance the chemotactic effect by elastin and fibronectin. The inflammatory response affects the pulmonary artery pressure mainly by changing the thickness of the endovascular intima, middle membrane and outer membrane^16^. The lymphocyte ratio decreased suggested an increased inflammatory response. Voelke *et al*. indicated that the lack of Treg cells caused the formation of pulmonary hypertension^17^. The study indicates that the increase of the ratio of neutrophils and lymphocytes in peripheral blood indicates that the prognosis of pulmonary hypertension patients is not good, and the need for lung transplantation treatment is greater^18^. Therefore, many studies emphasize that the treatment of inflammation in patients with pulmonary hypertension can improve the prognosis.

### Hypoxia is another cause of pulmonary hypertension

RDW is a parameter which reflects the volume heterogeneity of peripheral blood cells. Increased RDW indicates anemia, hematopoietic abnormalities, or congenital erythrocyte abnormalities. The serum RDW level in the pulmonary hypertension group was significantly higher than that in the control group^19^. The high RDW indicated that PH patient accompanied with anemia, and the change trend was same with hemoglobin concentration in Table 3, while there was no significant difference in the level of erythrocyte and hemoglobin in peripheral blood (*P* > 0.05). Anemia means that oxygenated hemoglobin level is reduced, and the body is in anoxic state. The difference in the oxygen saturation of the fingertip in Table 1 illustrated this point. Elevated RDW promotes the development of cardiovascular disease through chronic hypoxia, inflammatory response and oxidative stress^20^. Moreover, high RDW indicates poor prognosis in patients with pulmonary hypertension^18^. The oxygen saturation in the pulmonary hypertension group was significantly lower than that in the control group (*P* < 0.05), which was related to the “external environment” of the patient’s own disease and hypoxia in high altitude area. The level of serum ROCK2 in the pulmonary hypertension group was significantly higher than that in the non-pulmonary hypertension group (*P* < 0.05), which was consistent with previous studies showing that the Rho/rho-kinase pathway in low oxygen conditions was activated to participate in pulmonary hypertension. Existing studies have shown that activation of ROCK can be phosphorylated 653 serine of NHE1 on the pulmonary vascular smooth muscle cell membrane to alkaline the cell to promote proliferation and migration of pulmonary vascular smooth muscle cell^21,22^. Spearman correlation analysis was further conducted betweeen SpO2 and ROCK2 concentration, with r −0.307(p < 0.05), suggesting the lower SpO2,the higher ROCK2 level.Another under hypoxic condition, HIF-1 α expression increased, chronic hypoxia and inflammatory reaction influenced each other, and which can promote pulmonary vascular remodeling and thus affected the pulmonary arterial pressure^17^.

### Chronic liver dysfunction is common in patients with pulmonary hypertension

The late manifestation of pulmonary arterial hypertension is that chronic liver function injury caused by right cardiac dysfunction, which is associated with elevated hepatic venous pressure, inadequate perfusion, and insufficient oxygen supply^23^.

Serum bilirubin in the pulmonary hypertension group was significantly higher than that in the control group (*P* < 0.05), which confirmed that there was no significant difference between the two groups of serum glutamate transaminase and glutamate transaminase in Table 4. Recent studies have shown that hyperbilirubinemia is an important factor affecting the survival of patients with pulmonary hypertension, with a value of 23.7 umol/L^24^. The serum total protein and albumin level were significantly lower in pulmonary hypertension group (*P* < 0.05), which was associated with the ability of hepatic synthetic proteins and water sodium retention.

### Pulmonary hypertension and renal dysfunction affect each other

As shown in Table 5, renal perfusion in pulmonary hypertension group was insufficient, renal function deteriorated, excretion decreased, and CysC level increased. Wei Cai etc. Studies have shown that high uric acid hematic disease can reduce the NO level through the eNOs, and which intensified inflammation to damage endothelial cell functions by promoting the expression of IL - 6, TNF - α, ICAM-1, VCAM-1^25^. In this way, pulmonary arterial hypertension is caused by pulmonary vasoconstriction and remodeling^26^. Research confirmed that hyperuricemia and nitrogen qualitative hematic disease are independent predictors for prognosis of patients with pulmonary hypertension, as well as hyperuricemia is an independent risk factor for various cardiovascular diseases such as atherosclerosis^25,27,28^, so it is necessary to correct hyperuricemia. high uric acid hematic disease, including atherosclerosis, a variety of independent risk factors for cardiovascular disease^25^, so it is necessary to correct high uric acid hematic disease. In short, pulmonary hypertension leads to hyperuricemia, and hyperuricemia can in turn promote pulmonary hypertension.

## Conclusion

ROCK2 plays a leading role in pulmonary hypertension in the plateau region, so selective ROCK2 inhibitors will be more effective in improving pulmonary hypertension. Qinghai as a high-altitude area, the unique low oxygen environment can cause inflammation, which in turn can aggravate hypoxia, both of which can promote the occurrence of pulmonary hypertension. Oxygen therapy and improving inflammation should be taken seriously. In patients with pulmonary hypertension, right ventricular failure causes abnormal liver and kidney function, and hyperuricemia can promote the increase of pulmonary arterial pressure. Hyperbilirubinemia, hyperuricemia and hyperazemia indicate poor prognosis in patients with pulmonary hypertension.

## Materials and Methods

151 patients were included in the study, who were admitted to the departments of cardiovascular medicine or respiration medicine in Qinghai Province Peoples’ Hospital during December 2016 to June 2017, and they were long settled in Qinghai. This work has been approved by ethnic committee of Qinghai Provincial People’s Hospital. The subjects should exclude whose combine with cerebral infarction, cerebral hemorrhage, cerebral ischemia, central nervous system demyelinating disease, Parkinson’s disease, pancreatitis, bronchial asthma, malignant tumor, reactive rhinitis, etc. The group was based on the post-admission heart color doppler flow rate peak or pulmonary artery systolic blood pressure. There were 87 cases in the control group, including 47 males (54.02%) and 40 females (45.98%). The pulmonary hypertension group included 64 cases, including 34 males (53.13%) and 30 females (46.88%). The gender composition of the two groups was no difference (*P* > 0.05). The Han group in the control group included 54 cases (62.07%) and 33 cases of non-Han nationality (37.93%), while in the pulmonary hypertension group included 41 Han Nationality cases (64.06%) and 23 non-Han cases (35.94%). The ethnic composition of the two groups was no difference (*P* > 0.05). Control group included 35 hypertension cases (40.23%), 25 coronary heart disease cases (17.24%), 5 dilated cardiomyopathy cases (5.75%), 9 atrial fibrillation cases (10.34%), 9 chronic cor pulmonale cases (10.34%), 5 congenital heart disease cases (5.75%), 2 valvular heart disease cases (2.30%), and other 7 cases (8.05%). Pulmonary hypertension group included 33 chronic cor pulmonale patients (51.56%), 9 coronary heart disease cases (14.06%), 2 dilated cardiomyopathy cases (3.13%), 3 congenital heart disease cases (4.69%), 5 valvular heart disease cases (7.81%), 3 hypertension cases (4.69%), 3 atrial fibrillation cases (4.69%), 2 pulmonary embolism cases (3.13%), other 2 cases (3.13%).

The gender, age, ethnicity, height, weight, body mass index, height, residence time, blood pressure and fingertip oxygen saturation of each candidate were recorded (Table 1).

The disease composition of two groups were recorded (Table 2) and diagnosis was according to the latest guidelines.

The various indexes of cardiac color ultrasound (Table 3) were diagnosed by the experienced doctors in Qinghai Provincial People’s Hospital using the Philups iE33 color doppler ultrasound diagnostic instrument with a 1–5 MHz probe frequency.

Blood routine index (Table 4), liver and kidney function index (Tables 5 and 6) of all patients were measured by experienced doctors of Qinghai Provincial People’s Hospital using the blood routine analyzer of Japan’s Sysmex brand (XN-2000) and the automatic biochemical analyzer BECKMAN COULTER (AU5831). The data was carefully calibrated before the report.

We collected 87 cases of control group and 64 cases of pulmonary hypertension group fasting venous blood. After centrifugation, the upper serum was taken and stored in −80 °C refrigerator. The low speed centrifuge purchased from Anhui Zhongjia science instrument (KDC-1044) with a centrifugal speed of 3500/min and a time limit of 5 minutes.

Serum ROCK1 and ROCK2 were measured by enzyme-linked immunoassay. The ROCK1 enzyme-linked immunoassay kit was purchased from Shanghai Jining biotechnology co., LTD., and the sensitivity was 0.1 pg/ml, and the monitoring range was 2.5–80 ng/ml. The ROCK2 enzyme-linked immunoassay kit was purchased from Xiamen Kangyan biotechnology co., LTD., with a sensitivity of 7.8 pg/ml and a monitoring range of 31.2 to 2000 pg/ml.

The statistical analysis using SPSS 21.0 software. For the normal distribution measurement data, the results were expressed as mean ± standard deviation (*x* ± *s*) using an independent sample t test, while for the skewed distribution measurement data, the results were expressed as median (M) and quartile (P25, P75) with mann-whitney U test. The counting data used chi-square test. Values of *P* < 0.05 were considered statistically significant.

### Ethics approval and consent to participate

Written informed consent was obtained from each patient prior to enrolment, and the present study was approved by the ethics committee of Qinghai Province People’s Hospita.

## References

[1] Armstrong DW, Tsimiklis G, Matangi MF (2010). Factors influencing the echocardiographic estimate of right ventricular systolic pressure in normal patients and clinically relevant ranges according to age. Canadian Journal of Cardiology..

[2] Listed N (2009). Developing new guidelines for the diagnosis, and treatment of pulmonary hypertension. European Heart Journal..

[3] Nazzareno G (2009). ESC/ERS Guidelines for the diagnosis and treatment of pulmonary hypertension. European Heart Journal..

[4] Hartmann S, Ridley AJ, Lutz S (2015). The Function of Rho-Associated Kinases ROCK1 and ROCK2 in the Pathogenesis of Cardiovascular Disease. Frontiers in Pharmacology..

[5] Julian L, Olson MF (2014). Rho-associated coiled-coil containing kinases (ROCK). Small Gtpases..

[6] Michelle S (2014). ROCK1 deficiency enhances protective effects of antioxidants against apoptosis and cell detachment. Plos One..

[7] Lopez NC (2016). Role of the Rhoa/Rock pathway in high-altitude associated neonatal pulmonary hypertension in lambs. Am J Physiol Regul Integr Comp Physiol..

[8] Resta TC, Broughton BR, Jernigan NL (2010). Reactive oxygen species and RhoA signaling in vascular smooth muscle: role in chronic hypoxia-induced pulmonary hypertension. Advances in Experimental Medicine & Biology..

[9] Lin Y (2013). Prevention of diabetes-induced arginase activation and vascular dysfunction by Rho kinase (ROCK) knockout. Cardiovascular Research..

[10] Yao L (2010). The role of RhoA/Rho kinase pathway in endothelial dysfunction. Journal of Cardiovascular Disease Research..

[11] Wang Y (2009). ROCK isoform regulation of myosin phosphatase and contractility in vascular smooth muscle cells. Circulation Research..

[12] Xu EZ (2010). Rescue treatment with a Rho-kinase inhibitor normalizes right ventricular function and reverses remodeling in juvenile rats with chronic pulmonary hypertension. American Journal of Physiology Heart & Circulatory Physiology..

[13] Shimizu T (2013). Crucial role of ROCK2 in vascular smooth muscle cells for hypoxia-induced pulmonary hypertension in mice. Journal of Cardiac Failure..

[14] Feng Q (2016). ROCK2 mediates the proliferation of pulmonary arterial endothelial cells induced by hypoxia in the development of pulmonary arterial hypertension. Experimental & Therapeutic Medicine..

[15] Shimizu T (2017). Fibroblast deletion of ROCK2 attenuates cardiac hypertrophy, fibrosis, and diastolic dysfunction. Jci Insight..

[16] Rabinovitch M (2014). Inflammation and immunity in the pathogenesis of pulmonary arterial hypertension. Circulation Research..

[17] Voelkel NF, Tamosiuniene R, Nicolls MR (2016). Challenges and opportunities in treating inflammation associated with pulmonary hypertension. Expert Review of Cardiovascular Therapy..

[18] Harbaum L (2017). Exploratory analysis of the neutrophil to lymphocyte ratio in patients with pulmonary arterial hypertension. Bmc Pulmonary Medicine..

[19] Mazzotta E (2016). Red blood cell distribution width, hematology, and serum biochemistry in dogs with echocardiographically estimated precapillary and postcapillary pulmonary arterial hypertension. Journal of Veterinary Internal Medicine..

[20] Mozos I (2015). Mechanisms linking red blood cell disorders and cardiovascular diseases. Biomed Research International..

[21] Walker J (2016). Role of Rho kinase and Na+/H+ exchange in hypoxia-induced pulmonary arterial smooth muscle cell proliferation and migration. Physiological Reports..

[22] Wallert MA (2015). RhoA kinase (Rock) and p90 Ribosomal S6 kinase (p90Rsk) phosphorylation of the sodium hydrogen exchanger (NHE1) is required for lysophosphatidic acid-induced transport, cytoskeletal organization and migration. Cellular Signalling..

[23] Nikolaou M, Mebazaa A (2017). Cardiohepatic interactions in heart failure: clinical and therapeutic implications. Continuing Cardiology Education..

[24] Gong JN (2015). Serum bilirubin and 6-min walk distance as prognostic predictors for inoperable chronic thromboembolic pulmonary hypertension: a prospective cohort study. National Medical Journal of China..

[25] Cai W (2017). Uric acid induces endothelial dysfunction by activating the HMGB1/RAGE signaling pathway. Biomed Research International..

[26] Nickel NP (2017). Kidney dysfunction in patients with pulmonary arterial hypertension. Pulmonary Circulation..

[27] Kaiser R (2014). Prognostic impact of renal function in precapillary pulmonary hypertension. Journal of Internal Medicine..

[28] Dhaun N (2014). Endothelin antagonism and uric acid levels in pulmonary arterial hypertension: clinical associations. Journal of Heart & Lung Transplantation the Official Publication of the International Society for Heart Transplantation..

